# Lipoteichoic acid may affect the pathogenesis of PBC-like bile duct damage and might be involved in systemic multifocal epithelial inflammations in chronic colitis-harboring TCRα^−/−^ × AIM^−/−^ mice

**DOI:** 10.1080/08916930701402392

**Published:** 2007-06-22

**Authors:** Ikuko Haruta, Etsuko Hashimoto, Noriyuki Shibata, Yoichiro Kato, Makio Kobayashi, Keiko Shiratori

**Affiliations:** 1 Department of Medicine and Gastroenterology, Tokyo Women's Medical University, Tokyo, Japan; 2 Department of Pathology, Tokyo Women's Medical University, Tokyo, Japan

**Keywords:** TCRα^−/−^ mice, LTA, primary biliary cirrhosis, primary sclerosing cholangitis, multiple organ dysfunction syndrome (MODS)

## Abstract

**Background:**

Chronic colitis-harboring TCRα^−/−^ × AIM^−/−^ mice showed PBC-like bile duct damage in the liver. Bacterial infection is one of the candidates for the pathogenesis of PBC. We demonstrated that the bacterial cell wall component lipotheicoic acid (LTA)was detected at sites of inflammation around damaged bile ducts in PBC patients. The aim of this study was to investigate the pathophysiology of the liver and other organs in TCRα^−/−^ × AIM^−/−^ mice.

**Methods:**

Thirteen female TCRα^−/−^ × AIM^−/−^ mice were sacrificed at 24 weeks of age. The liver, stomach, small intestine, colon, pancreas, kidney and spleen were studied for pathological examination. Using anti-LTA antibody as the primary antibody,an immunohistochemical study was carried out.

**Results:**

In the liver, LTA was mainly detected in the portal area with inflammation, and some of the cytoplasm of hepatocytes. Inflammations were also observed in the stomach, intestine, pancreas and kidney. Throughout the gastrointestinal tract, from the stomach to the colon, LTA was detected in the epithelium at sites of inflammation. Furthermore, LTA was detected around both pancreatic ducts with inflammation and distal renal tubules with inflammation.

**Conclusions:**

The development of inflammations in the liver as well as extensive organs, strongly suggests a close relationship between bile duct damage and systemic multifocal epithelial inflammations, perhaps involving bacterial LTA, in TCRα^−/−^ × AIM^−/−^ mice.

## Introduction

Intrahepatic bile ducts are targets for inflammation in both primary biliary cirrhosis (PBC) and primary sclerosing cholangitis (PSC), yet the mechanisms of biliary epithelial cell damage in these diseases are not clearly understood. We previously reported that defects in apoptosis inhibitor expressed by macrophages (AIM) affected portal inflammation as well as biliary epithelial cell damage in the liver in colitis-harboring female TCRα-deficient (TCRα^−/−^) mice [1]. Although the TCRα^−/−^ mouse is an ulcerative colitis (UC)-like colitis-harboring mouse,the pattern of bile duct damage was closer to PBC in TCRα^−/−^ × AIM −/− double-knockout mice.

AIM is an apoptosis inhibitory factor belonging to the macrophage scavenger receptor cystein-rich superfamily (SRCR-SF), which is solely secreted by tissue macrophages [2]. SRCR-SF members play important roles in the uptake and clearance of effete components, such as modified host molecules and apoptotic cells [3,4]. Recently, Arai et al. reported that AIM (same as Spα/Api6) expression was under the regulation of liver X receptors (LXR)/retinoid X receptors (RXR) in macrophages [5]. Joseph et al. reported that the LXR target gene, Spα (i.e. AIM), was induced during *Listeria monocytogenes* infection [6]. LXRs are positive regulators of the SRCR family member SPα. LXR-dependent gene expression plays various roles in innate immunity. Valledor et al. showed that common nuclear receptor transcriptional pathways may be utilized to facilitate the clearance of apoptotic cells and bacterial pathogens, and that bacterial infection induces AIM expression via LXR/RXR activation [7].

Recently, the relationship between bacteria and the pathogenesis of PBC [8], in particular enterobacterial antigens [9], has been reported. Tuneyama et al. reported that the bacterial cell wall component lipotheicoic acid (LTA) was detected around the portal tract and sinusoidal cells [10]. We also reported that LTA was detected at the portal tract in stage 1–2 PBC with chronic non-suppurative destructive cholangitis (CNSDC). In our observation, the pattern of LTA localization depends on the pathological stage, with marked differences between stages [11]. On this basis, we speculated that LTA might affect the pathogenesis of the inflammatory cellular infiltration and focal disruption of the basement membrane in the portal area in TCRα^−/−^ × AIM^−/−^ mice. The aim of the present study was to investigate the effect of LTA on bile duct damage in TCRα^−/−^ × AIM^−/−^ mice. Furthermore, we investigated the pathological alteration and localization of LTA in extensive organs in these TCRα^−/−^ × AIM^−/−^ mice. From the aspect of systemic multifocal organ inflammation, the role(s) of LTA in the initiation and/or progression of inflammation, not only in the liver but also in other organs of these TCRα^−/−^ × AIM^−/−^ mice, might be of interest.

## Materials and methods

### Mice

TCRα^−/−^ mice were from a mixed C57BL/6J × 129 genetic background and obtained from The Jackson Laboratory (Bar Harbor, ME, USA). TCRα^−/−^ × AIM^−/−^ mice were generated in the Basel Institute of Immunology (Basel, Switzerland) by interbreeding of TCRα^−/−^ and AIM^−/−^ single-gene-deficient mice (kind gift from Dr Miyazaki).Mice were bred and maintained in a specific pathogen-free animal facility at the Basel Institute for Immunology until about 8 weeks of age. They were then transferred and maintained in a semi-specific pathogen-free animal facility at the Tokyo Women's Medical University (Tokyo, Japan). All animal experiments were approved by the Research Ethics Committee of Tokyo Women's Medical University [1].

### Tissue preparation

Female mice were used for the experiments. Thirteen TCRα^−/−^ × AIM^−/−^ mice at 24 weeks of age were used for the experiments. Mice were sacrificed under deep anesthesia using diethyl ether. Liver, stomach, small intestine, colon, pancreas, kidney and spleen tissues were taken, fixed in 10% formalin, and embedded in paraffin. Multiple 4-μm-thick sections cut from the formalin-fixed, paraffin-embedded tissues were used for pathological examinations. As for the control, wild-type (TCRα^+/+^ × AIM^−/−^ ,i.e. C57BL/6J) mice liver was studied for immunochemical staining.

### Pathology

Sections were stained with hematoxylin–eosin (H&E) for light microscopic examination. Serial sections were subjected to immunohistochemical staining with polyclonal rabbit anti-LTA antibody as the primary antibody (Biogenesis Inc., Kingston, NH, USA) at 1:500 dilution. These sections were then stained by a peroxidase technique using a Vectastain Elite ABC Kit (VectorLab., Burlingame, CA, USA) following the manufacturer's instructions. These sections were then stained with hematoxylin [11].

## Results

### Pathological findings

#### Liver

**Figure 1:**
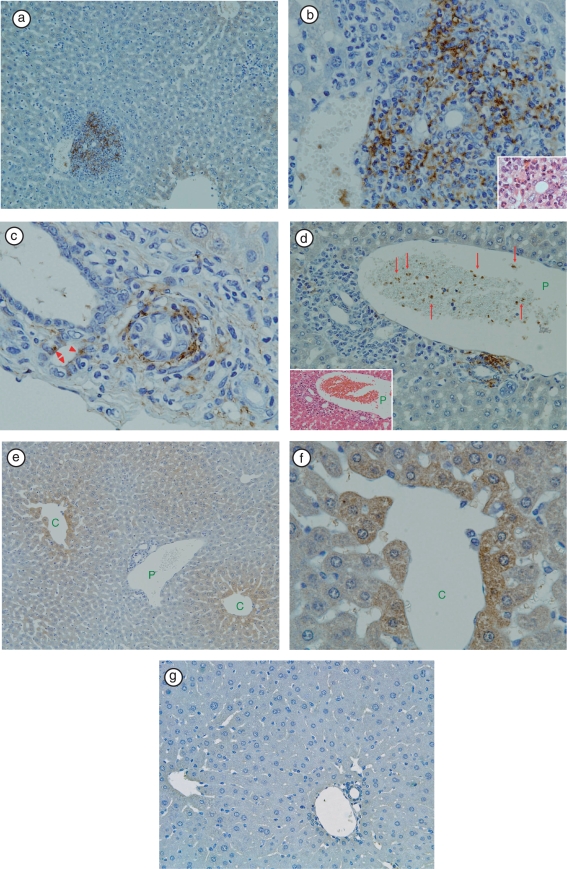
Immunohistochemical staining of LTA in TCRα^−/−^ × AIM^−/−^ mouse liver. (a) At a lower magnification, LTA immunoreactivity was prominent in the portal areas, but not in hepatic sinusoidal cells.(b) In the same sample, at ahigher magnification of the portal area, LTA immunoreactivity was observed in the cytoplasm of polymorphic inflammatory cells. H&E staining of the same bile duct is shown in the right lower corner. (c) In another TCRα^−/−^ × AIM^−/−^ mouse liver, LTA was detected in the cytoplasm of bile duct epithelial cells (arrowheads), and connective tissue of Glisson's sheath. (d) The left lower corner shows H&E staining of the portal tract. Red blood cells and white blood cells can be observed in the portal vein branch. (P: portal vein). Amorphous materials were intermingled with blood cells in the lumens of the portal vein branch (arrows). (e) In the hepatic lobules, LTA was mainly located in the hepatocytes adjacent to the central vein. (C: central vein, P: portal vein). (f) In the same sample, at a higher magnification of the hepatic lobules, LTA localizations were clearly observed each of the single hepatocytes (C: central vein).(g) LTA immunoreactivity was not detectable in wild-type (C57BL/6J) mice liver.

As we previously reported, necroinflammatory changes in hepatic lobules were subtle [1]. LTA immunoreactivity was detectable in the livers of 5 of 13 (38.4%) TCRα^−/−^ × AIM^−/−^ mice. At a lower magnification, immunoreactivity was prominent in the portal areas, but not in hepatic sinusoidal cells (Figure 1a). At a higher magnification of the portal area, LTA immunoreactivity was observed in the cytoplasm of polymorphic inflammatory cells (Figure 1b), in the cytoplasm of bile duct epithelial cells (Figure 1c), in amorphous materials intermingled with blood cells in the lumens of the portal vein branches (Figure 1d), and in the connective tissue of Glisson's sheath (Figure 1c). At a higher magnification of the hepatic lobules, LTA was mainly located in the hepatocytes adjacent to the central veins (Figure 1e,f). LTA immunoreactivity was not detectable in 3 of 3 wild-type (C57BL/6J) mice liver (Figure 1g).

#### Stomach

**Figure 2:**
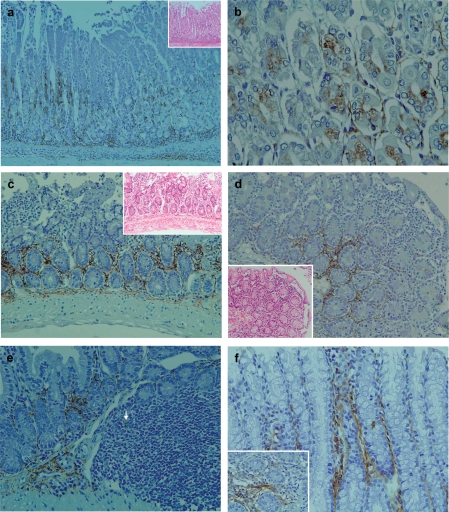
Pathological findings and immunohistochemical staining of LTA in the gastrointestinal tract of TCRα^−/−^ × AIM^−/−^ mice. (a) In the stomach, mild polymorphic inflammatory cellular infiltrates were observed in both mucosal and submucosal layers in all of the TCRα^−/−^ × AIM^−/−^ mice. LTA immunoreactivity was detectable in the proper mucosal layer. LTA was localized in the cytoplasm of both the fundic gland epithelial cells at the basal regions and polymorphic inflammatory cells. In the submucosal layer, immunoreactivity to LTA was observed in the cytoplasm of inflammatory cells and the stromal connective tissues. (b) At a higher magnification, LTA was localized in the cytoplasm of both the fundic gland epithelial cells at the basal regions.(c) In the small intestines of TCRα^−/−^ × AIM^−/−^ mice, mild to moderate polymorphic inflammatory cellular infiltrates were observed in the mucosal layers (H&E, on the higher right corner). In the proper mucosal layer, LTA immunoreactivity was located in the cytoplasm of inflammatory cells at the basal lesions.(d) In another TCRα^−/−^ × AIM^−/−^ mouse, mild to moderate polymorphic inflammatorycellular infiltrates were observed in the mucosal layers of the small intestine (H&E, on the lower left corner). In the proper mucosal layer, LTA immunoreactivity was located in the cytoplasm of inflammatory cells at the basal lesions. (e) In the submucosal layer, LTA immunoreactivity was observed in the connective tissue and in the cytoplasm of some of the inflammatory cells (arrow) in Peyer's patches. (f) In the colon, LTA immunoreactivity was detectable in the colon of all TCRα^−/−^ × AIM^−/−^ mice. LTA was detected in the cytoplasm of mucosal inflammatory cells and in the connective tissue of the proper mucosal layer.

In the stomach, mild polymorphic inflammatory cellular infiltrates were observed both in mucosal and submucosal layers in all TCRα^−/−^ × AIM^−/−^ mice (Figure 2a). LTA immunoreactivity was detectable in the proper mucosal layer of all TCRα^−/−^ × AIM^−/−^ mice. LTA was localized in the cytoplasm of both the fundic gland epithelial cells at the basal regions (Figure 2a,b) and polymorphic inflammatory cells. In the submucosal layer, immunoreactivity to LTA was observed in the cytoplasm of inflammatory cells and the stromal connective tissues (Figure 2a).

#### Small intestine

In the small intestine, mild to moderate polymorphic inflammatory cellular infiltrates were observed in mucosal layers of all TCRα^−/−^ × AIM^−/−^ mice (Figure 2c,d). LTA immunoreactivity was detectable in the small intestine of all TCRα^−/−^ × AIM^−/−^ mice. In the proper mucosal layer, LTA immunoreactivity was located in the cytoplasm of inflammatory cells at the basal lesions. In the submucosal layer, LTA immunoreactivity was observed in the connective tissue and in the cytoplasm of some inflammatory cellsin Peyer's patches (Figure 2e).

#### Colon

In the colon, severe colitis was observed in all TCRα^−/−^ × AIM^−/−^ mice [1]. LTA immunoreactivity was detectable in the cytoplasm of mucosal inflammatory cells in all TCRα^−/−^ × AIM^−/−^ mice (Figure 2f).

#### Pancreas

**Figure 3:**
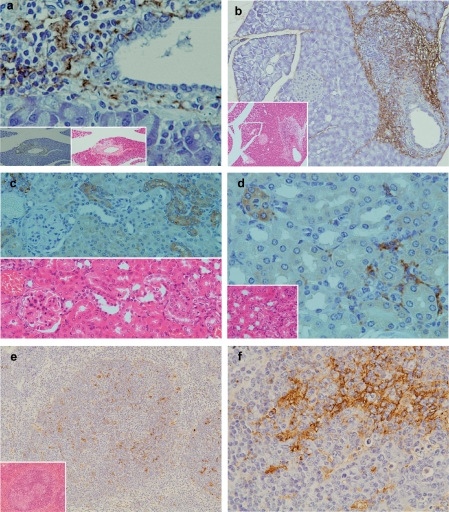
Pathological findings and immunohistochemical staining of LTA in the pancreas, kidney and spleen. (a) In the pancreas, marked inflammatory cellular infiltrates around pancreatic ducts were observed (H&E, lower middle column). LTA was located in the cytoplasm of polymorphic inflammatory cells and in the stromal connective tissue around pancreatic ducts (lower magnification in the left lower corner). (b) In another TCRα^−/−^ × AIM^−/−^ mouse, marked inflammatory cellular infiltrates around pancreatic ducts were observed (H&E, lower left corner). LTA was also detected in the cytoplasm of polymorphic inflammatory cells and the stromal connective tissue around pancreatic ducts (immunohistochemistry). (c) In the kidney, mild inflammatory cellular infiltrates around renal tubuli were observed (H&E, lower left corner). LTA immunoreactivity was detected in the cytoplasm of polymorphic inflammatory cells, and in the stromal connective tissues of the cortex. (d) In another TCRα^−/−^ × AIM^−/−^ mouse, LTA immunoreactivity was detected in the cytoplasm of some distal renal tubuli. Lower half shows H&E staining of the sequential sliced section. (e) LTA immunoreactivity was mainly detected in the cytoplasm of lymphocytes in the white pulp of the spleen. Lower left corner shows H&E staining. (f) In the same sample as (e), at a higher magnification of the spleen, LTA immunoreactivity was mainly detected in the cytoplasm of lymphocytes in the white pulp.

In the pancreas, marked inflammatory cellular infiltrates around pancretatic ducts were observed in 4 of the 13 (30.7%) TCRα^−/−^× AIM^−/−^ mice. Inflammatory cells around pancreatic ducts consisted mainly of neutrophils and a fewly mphocytes (Figure 3a,b). LTA immunoreactivity was detectable in the pancreas of TCRα^−/−^ × AIM^−/−^ mice. LTA was located in the cytoplasm of polymorphic inflammatory cells and the stromal connective tissue around pancreatic ducts (Figure 3a,b).

#### Kidney

**Figure 4:**
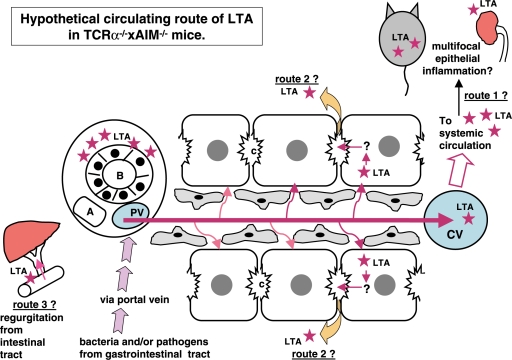
Hypothetical circulating route of LTA in TCRα^−/−^ × AIM^−/−^ mice. At first, bacteria and/or pathogens including LTA were taken from the epithelia of the gastrointestinal tract. LTA and/or bacterial components,as well as other pathogens, reached the liver via portal blood flow. These LTA and/or LTA-containing materials would be drained toward the central vein in the liver. LTA immunoreactivities were detectable not only in the liver and gastrointestinal tract, but also in the pancreas, kidney, and spleen. Via systemic blood flow, LTA could be trapped in other organs, which might possess a higher affinity to LTA (speculative route 1). LTA was also detected within the cytoplasm of hepatocytes. Perhaps LTA that escaped to be phagocitized by Kupffer cells is taken into hepatocytes via sinusoidal blood flow from the space of Disse (speculative route 2). There are three possible explanations for how LTA accumulates around the interlobular bile duct. One speculation is that from the hepatocytes, via bile canaliculi to Herring's canal, LTA was secreted and finally reached the interlobular bile ducts. The other speculation is that, because of regurgitation from intestinal tract to bile ducts, LTA finally reached the interlobular bile ducts (speculative route 3). The third speculation is that, may be because of the high affinity of bile ducts to LTA, LTA accumulates around bile ducts via systemic arterial blood flow throughout route 1. After reaching the bile ducts, leakage of LTA around bile ducts would occur.

LTA was detectable in the kidneys of 5 of 13 (38.4%) TCRα^−/−^ × AIM^−/−^ mice, which showed mild inflammatory cellular infiltrates around the renal tubules (Figure 4c). LTA immunoreactivity was detected in the cytoplasm of epithelial cells of some distal renal tubules (Figure 4c,d), polymorphic inflammatory cells (Figure 4d), and in the stromal connective tissues of the cortex (Figure 4d).

#### Spleen

LTA immunoreactivity was detectable in the spleens of all TCRα^−/−^ × AIM^−/−^ mice. LTA immunoreactivity was mainly detected in the cytoplasm of lymphocytes in white pulp (Figure 3e,f).

## Discussion

In the present study, we first demonstrated that in TCRα^−/−^ × AIM^−/−^ mice, a marked accumulation of LTA was observed in the liver, especially the portal area with inflammations (Figure 1a–f), as in stage 1–2 human PBC liver [11]. LTA has been considered a surface reactive antigen and/or agent that mediates the attachment of certain bacteria to host tissues [11–13]. Based on these observations, we speculated that LTA might affect the initiation and/or progression of bile duct damage in TCRα^−/−^ × AIM^−/−^ mice, as in early-stage PBC.

Second, we demonstrated that the inflammation was observed not only in the liver and the colon [1], but also in the stomach, small intestine, pancreas and kidney (Figures 2 and 3). Throughout these organs, it appeared that the epithelium was the major target organ for inflammations in TCRα^−/−^ × AIM^−/−^ mice. It has been reported that autoimmune pancreatitis (AIP), which is characterized by a collar of periductal inflammation histologically [14], is occasionally observed in association with PBC, Sjögren's syndrome, and PSC [15]. The findings observed in TCRα^−/−^ × AIM^−/−^ mice could be a kind of multiple-organ inflammation. Interestingly, at the sites of inflammation, LTA was accumulated mainly around the epithelium (Figures 2 and 3). AIM is thought to contribute to the clearance of dead cells, as well as toxic reagents including bacteria themselves or LPS and soon by macrophages [3,4]. The lack of AIM in TCRα^−/−^ × AIM^−/−^ mice might result in a more persistent localization of toxic reagents, perhaps including LTA, at inflammatorysites.

Throughout these studies, the origin of LTA, i.e. identification of the Gram-positive organisms release it, was not determined. Gram-positive bacteria such as *Propionibacterium acnes*, *Staphylococcus aureus*, *Enter-ococcus faecalis*, *Bacillus subtilis*, *Streptococcus pyogenes* and so on contain LTA [16,17]. Out of these, *P. acnes* DNA is present as a major clone in granulomas of PBC livers [18]. *E. faecalis* could cause urinary tract infections [19]. Although we did not test fecal and urinary culture in the present study, their examination in TCRα^−/−^ × AIM^−/−^ mice might be expected to provide some additional information.

De Kimpe et al. reported that the Gram-positive bacterial component LTA can induce inflammatory responses and multiple organ dysfunction syndrome (MODS) in rats, together with peptidoglycan (PepG). LTA and PepG from *S. aureus* act together to elicit widespread systemic inflammation in the liver, kidney, pancreas, heart and skeletal muscle. However, LTA or PepG alone caused minor induction of inflammation without MODS. In contrast to LPS, LTA or PepG does not cause lethality or organ toxity at an immunostimulatory dose in mice or rats [20]. Although we did not examine the regulation of PepG in TCRα^−/−^ × AIM^−/−^ mice, we speculated that prolonged low-dose stimulation by LTA could cause not immediately lethal but chronic inflammation, not only in the portal tract in the liver but also in a variety of organs in the abdomens of TCRα^−/−^ × AIM^−/−^ mice.

We then, hypothesized regarding the possible circulating route(s) of LTA. As shown in Figure 4, at first, bacteria and/or pathogens including LTA were taken from the epithelia of the gastrointestinal tract. LTA and/or bacterial components, as well as other pathogens, reached the liver via portal blood flow. These LTA and/or LTA-containing materials would be drained toward the central vein in the liver. LTA immunoreactivities were detectable not only in the liver and gastrointestinal tract, but also in the pancreas, kidney, and spleen. Although we did not study the serum levels of LTA in these TCRα^−/−^ × AIM^−/−^ mice, soluble LTA might affect the pathogenesis of multifocal inflammations in these mice. Our findings would indicate that via systemic blood flow, LTA could be trapped in other organs, which might possess a higher affinity to LTA (speculative route 1 in Figure 4). However, LTA was also detected within the cytoplasm of hepatocytes (Figure 1e,f). It has been reported that alcohol-induced liver injury involved an increase in circulating endotoxins, leading to activation of Kupffer cells [21]. In our study, LTA was not detected in Kupffer cells. Perhaps LTA that escaped to be phagocitized by Kupffer cells is taken into hepatocytes via sinusoidal blood flow from the space of Disse (speculative route 2 in Figure 4). Furthermore, LTA was detected within hepatocytes, which were mainly located around the central vein. Generally, drugs or toxins are thought to be metabolized to toxic intermediates predominantly by zone 3 hepatocytes [22]. It might be possible that the LTA pathway is similar to that of toxic metabolites, so that LTA-positive hepatocytes were detected around the central vein.

There are three possible explanations for how LTA accumulates around the interlobular bile duct. One speculation is that from the hepatocytes, via bile canaliculi to Herring's canal, LTA was secreted and finally reached the interlobular bile ducts. The other speculation is that, because of regurgitation from the intestinal tract to bile ducts, LTA finally reached the interlobularbile ducts(speculative route 3 in Figure 4). The third speculation is that, may be because of the high affinity of bile ducts to LTA, LTA accumulates around bile ducts via systemic arterial blood flow throughout route 1. After reaching the bile ducts, leakage of LTA around bile ducts would occur. Sakashita et al. described that substantial alteration in the tight junction may cause increased paracellular permeability in cholestatic disease, PBC [23]. It is not certain whether the accumulation of LTA causes the inflammation around bile ducts, or because of the inflammation, permeability of bile ducts are up-regulated allowing leakage of LTA to occur in these TCRα^−/−^ × AIM^−/−^ mice. To study the mechanism of accumulation of LTA around bile ducts would possibly provide us some insight into the bile duct damage as well as multifocal organ (mainly, epithelial) inflammation in TCRα^−/−^ × AIM^−/−^ mice.

In conclusion, LTA accumulation was observed in female TCRα^−/−^ × AIM^−/−^ mice livers, corresponding to portal inflammation and biliary epithelial cell damage, as in the human PBC liver. Inflammations in TCRα^−/−^ × AIM^−/−^ mice were observed not only in the colon and liver, but in various other organs as well. Furthermore, at the site of inflammations in these organs, marked accumulation of LTA was observed, as in the liver. AIM deficiency in TCRα^−/−^ mice would result in multifocal inflammation, perhaps in which LTA was involved. Studying the role of LTA in these TCRα^−/−^ × AIM^−/−^ mice might give us some insight into MODS in TCRα^−/−^ × AIM^−/−^ mice.
